# Toxicity-guided dose modification for disseminated *Nocardia farcinica* brain abscess in a patient with pneumoconiosis: a brief research report

**DOI:** 10.3389/fphar.2026.1805920

**Published:** 2026-04-09

**Authors:** Hui Juan Liu, Liang Fen Wang, Xiao Yue Li, Ling Li

**Affiliations:** 1 Department of Pharmacy, 363 Hospital, Chengdu, China; 2 Department of Neurosurgery, 363 Hospital, Chengdu, China; 3 Department of Clinical Laboratory, 363 Hospital, Chengdu, China

**Keywords:** antimicrobial stewardship, brain abscess, *Nocardia farcinica*, pneumoconiosis, toxicity-guided dosing, trimethoprim-sulfamethoxazole

## Abstract

**Background:**

Optimal antimicrobial strategies for disseminated nocardiosis with central nervous system (CNS) involvement remain poorly defined, particularly regarding trimethoprim-sulfamethoxazole (TMP-SMX) dosing in immunocompromised patients with severe drug intolerance.

**Methods:**

This observational case study analyzed the clinical course and pharmacological management of a 55-year-old male gold miner with pneumoconiosis and chronic corticosteroid use who developed *Nocardia farcinica* brain abscess. Diagnosis was established via metagenomic next-generation sequencing (mNGS) and phenotypic culture. An individualized antimicrobial regimen was designed based on toxicity monitoring.

**Results:**

Diagnosis of *N. farcinica* was confirmed by mNGS within 48 h. The patient initially failed empirical meropenem but responded to combination therapy with imipenem, amikacin, and TMP-SMX. Due to grade III gastrointestinal toxicity (CTCAE v5.0), TMP-SMX was de-escalated from 15 mg·kg^−1^·d^−1^–11.25 mg·kg^−1^·d^−1^, with maintenance at 7.5 mg·kg^−1^·d^−1^. Clinical improvement was observed at Day 120, though durable cure remains unconfirmed.

**Conclusion:**

In extreme circumstances of severe dose-limiting toxicity, temporary TMP-SMX dose reduction with intensive monitoring may be feasible as a bridge to complete guideline-concordant therapy, though this approach falls below current recommendations and requires robust therapeutic drug monitoring. Species-directed antimicrobial selection and early molecular diagnosis facilitated initial clinical resolution in this high-risk immunocompromised host.

## 1 Introduction

*Nocardia* species are aerobic, filamentous, Gram-positive actinomycetes that function as opportunistic pathogens, particularly in immunocompromised hosts. Among these, *Nocardia farcinica* demonstrates pronounced neurotropism; CNS invasion is associated with mortality rates reaching 55% in immunosuppressed populations (Rafiei et al., 2016; Soueges et al., 2022). Pneumoconiosis, characterized by progressive pulmonary fibrosis, impairs alveolar macrophage function and Th1-mediated cellular immunity. When superimposed upon systemic corticosteroid therapy, this creates a “dual-hit” immunodeficiency state that substantially amplifies susceptibility to invasive nocardiosis (Wang et al., 2023).

The management of CNS nocardiosis remains clinically challenging. Current guidelines recommend high-dose trimethoprim-sulfamethoxazole (TMP-SMX; ≥15 mg·kg^−1^·d^−1^ of the TMP component) as first-line therapy; however, this dosing paradigm lacks prospective validation and is frequently limited by hematologic or renal toxicity (Bodilsen et al., 2024).

A critical therapeutic dilemma emerges when severe, life-threatening TMP-SMX toxicity necessitates dose reduction during the treatment of disseminated CNS disease. Current guidelines offer no evidence-based recommendations for this scenario, leaving clinicians to balance the risk of treatment failure against drug-related mortality.

We hypothesized that in such exceptional circumstances, temporary toxicity-guided dose modification of TMP-SMX, when coupled with intensive clinical monitoring and synergistic combination therapy (imipenem + amikacin), may serve as a feasible bridging strategy to complete therapy without compromising outcomes.

To our knowledge, this represents the first reported case of pneumoconiosis complicated by disseminated *N. farcinica* brain abscess successfully managed with toxicity-guided TMP-SMX dose modification. While standard dosing remains the unequivocal goal (Bodilsen et al., 2024; Yetmar et al., 2025), this case contributes to the limited literature on management strategies when guideline-concordant dosing is intolerable due to severe adverse events. We emphasize that this approach constitutes a temporary risk-benefit trade-off in exceptional clinical scenarios, not a challenge to established standards of care.

## 2 Methods

### 2.1 Study design and ethics

This observational case study was conducted at 363 Hospital, Chengdu, China, with Ethics Committee approval and patient informed consent.

### 2.2 Diagnostic procedures

Brain abscess pus underwent Gram staining (1% sulfuric acid decolorizer), aerobic culture on blood agar (Chengdu Ruiqi Bio-technology, China) (35 °C, 5% CO_2_, 7 days), and mNGS. mNGS used QIAamp DNA Mini Kit (Qiagen) and BGISEQ-500 platform (BGI Genomics, Shenzhen, China) (50-bp paired-end, 20M reads), with bioinformatics filtering human sequences (hg19) and aligning to microbial databases.

### 2.3 Therapeutic intervention and monitoring

A multidisciplinary team (infectious diseases, pharmacy, and neurosurgery) directed antimicrobial therapy based on the patient’s actual body weight (66 kg): imipenem 500 mg every 6 h, amikacin 7.5 mg·kg^−1^ every 12 h, and TMP - SMX 15 mg·kg^−1^·d^−1^ of the TMP component in three divided doses. Amikacin *Note:* Divided dosing was selected over once-daily dosing (10–15 mg·kg^−1^) due to (1) the need to maintain consistent drug levels given amikacin’s poor blood-brain barrier penetration; (2) borderline renal function (eGFR 65 mL/min/1.73 m^2^); and (3) enhanced synergistic activity with β-lactams through more frequent dosing. We acknowledge that once-daily dosing per concentration-dependent pharmacokinetic/pharmacodynamic principles is preferred for most systemic infections.

Outcome Assessment and Safety Monitoring Primary outcome measures included clinical resolution of symptoms, radiological improvement on serial MRI, and management of adverse events. Toxicities were graded according to the Common Terminology Criteria for Adverse Events version 5.0 (CTCAE v5.0). Dose adjustments were based on clinical response, renal function monitoring (serum creatinine every 48–72 h), and patient tolerability.

As this is an observational single-case study, traditional biological replication and inferential statistical testing were not applicable. Microbiological identification was confirmed by two independent methods (culture and mNGS), and clinical assessments were adjudicated by the multidisciplinary team. Continuous variables are presented as absolute values with reference ranges; temporal trends use descriptive statistics from repeated measurements (Days 0–29).

## 3 Results

### 3.1 Patient characteristics and presentation

A 55-year-old male former gold miner (weight: 66 kg) with stage II silicosis and 10-year history of prednisone therapy (peak dose 30 mg·d^−1^) presented with progressive cough, purulent sputum, and dyspnea persisting for 1 month. Admission investigations demonstrated white blood cell count of 6.63 × 10^9^/L (reference range: 3.5–9.5 × 10^9^/L), and elevated C-reactive protein (184.28 mg/L; reference: <6.0 mg/L). Chest computed tomography confirmed left lower lobe consolidation and diffuse nodular infiltrates consistent with advanced pneumoconiosis. Admission chest CT findings are provided in Supplementary Figure S1. Laboratory findings supported immunodeficiency: lymphocyte count 0.60 × 10^9^/L (reference: 1.1–3.2 × 10^9^/L), lymphocyte proportion 8.6% (reference: 20%–50%), and positive cytomegalovirus IgG (>1000 AU/mL) indicating prior immunocompromised state. Laboratory investigations for tuberculosis, fungal pathogens, and influenza A/B virus were negative.

Immunological assessment revealed profound combined immunosuppression: (i) occupational pneumoconiosis (stage II silicosis, 10-year duration) with associated alveolar macrophage dysfunction and defective Th1-mediated cellular immunity; (ii) chronic corticosteroid therapy (prednisone equivalent 10–30 mg daily for 10 years). This “dual-hit” immunodeficiency state represents a high-risk category where disseminated nocardiosis carries mortality exceeding 50%, per current IDSA/ESCMID guidelines for CNS nocardiosis in immunocompromised hosts.

Concurrent pulmonary infection. Admission chest CT demonstrated left lower lobe consolidation with diffuse nodular infiltrates. Bronchoalveolar lavage fluid (BALF) obtained on Day 5 was culture-negative (likely due to prior piperacillin-tazobactam exposure). Pulmonary symptoms (cough, purulent sputum) resolved by Day 14 of targeted antimicrobial therapy. The concurrent pulmonary-CNS presentation is consistent with hematogenous dissemination from the primary pulmonary focus, a typical pattern in immunocompromised hosts with *N. farcinica* infection.

### 3.2 Clinical course and microbiological findings

**FIGURE 1:**
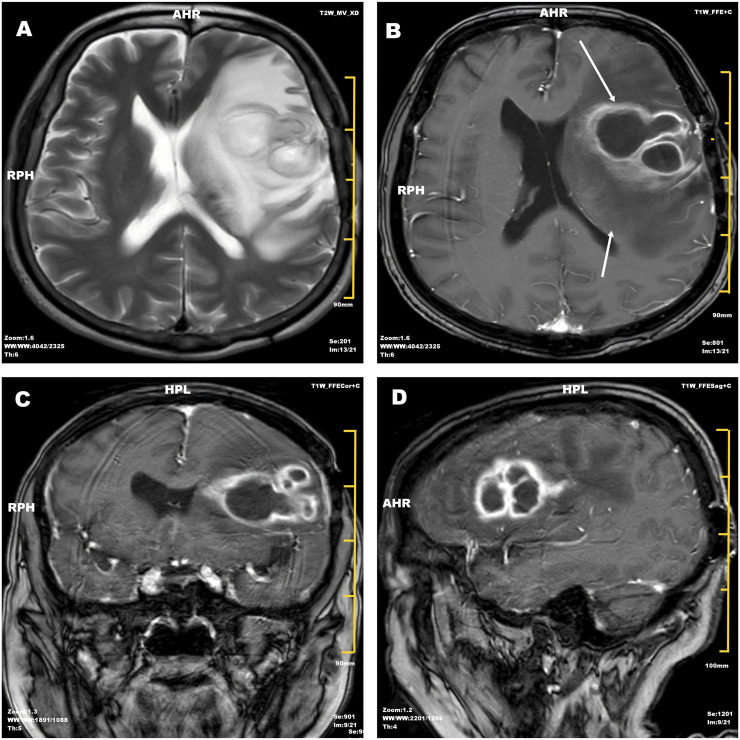
Cranial magnetic resonance imaging demonstrating brain abscess. **(A)** Diffusion-weighted imaging showing a 47 × 31 × 43 mm hyperintense lesion in the left frontal lobe with restricted diffusion. **(B)** T1-weighted post-contrast image revealing peripheral ring enhancement (arrowheads) with surrounding edema. **(C)** Apparent diffusion coefficient map demonstrating hypointense signal, confirming restricted diffusion. **(D)** Coronal FLAIR sequence showing mass effect with 6 mm midline shift. MRI: 3.0 T; gadolinium 0.1 mmol/kg IV.

Initial empirical therapy with piperacillin-tazobactam (4.5 g every 8 h) was initiated. On Day 9, neurological deterioration developed, including dysarthria, right facial droop, and generalized seizures, accompanied by fever (38.8 °C). Magnetic resonance imaging revealed a 47 × 31 × 43 mm lesion in the left frontal lobe with significant surrounding edema, consistent with brain abscess (Figure 1). Empirical antimicrobial coverage was broadened to vancomycin (1 g daily) and meropenem (2 g every 8 h). By Day 14, fever subsided, but neurological deficits persisted.

**FIGURE 2:**
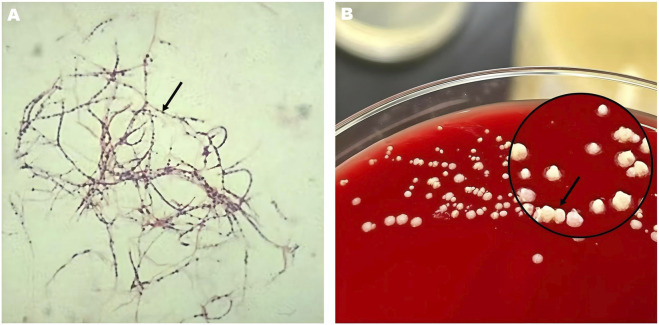
Microbiological identification of *Nocardia* farcinica. **(A)** Gram stain of abscess fluid (×1000, oil immersion) showing filamentous, branching Gram-positive rods with irregular beading (arrows). **(B)** Colony morphology on blood agar after 72 h at 35 °C demonstrating dry, chalky colonies with aerial hyphae, characteristic of N. farcinica.

On Day 15, craniotomy and drainage yielded approximately 40 mL of yellow purulent fluid. Gram staining demonstrated branching filamentous Gram-positive rods with irregular beading; modified acid-fast staining was weakly positive. After 72 h of incubation, culture revealed white, dry, chalky colonies with characteristic aerial hyphae (Figure 2). Metagenomic next-generation sequencing definitively identified *N. farcinica* within 48 h.

On Day 21, bacterial culture of the brain abscess fluid indicated *Nocardia* cutis. The isolate was reported as susceptible to TMP-SMX by phenotypic testing, though quantitative MIC data were unavailable due to lack of standardized susceptibility testing reagents at our institution. This limitation precludes pharmacokinetic/pharmacodynamic optimization based on MIC-guided dosing.

### 3.3 Pharmacological management and outcomes

Following pathogen identification, antimicrobial therapy was adjusted to imipenem (500 mg every 6 h), amikacin (7.5 mg·kg^−1^ intravenously every 12 h), and TMP-SMX (15 mg·kg^−1^·d^−1^ of the TMP component). Within 24 h, the patient developed grade III gastrointestinal toxicity (nausea and vomiting), necessitating dose interruption.

TMP-SMX was de-escalated to 11.25 mg·kg^−1^·d^−1^ and administered with meals (crushed tablets with food), resulting in rapid resolution of adverse events. This dose reduction was necessitated by life-threatening toxicity and represented a temporary risk-benefit trade-off, not an evidence-based dosing strategy. Imipenem and amikacin were continued.

**FIGURE 3:**
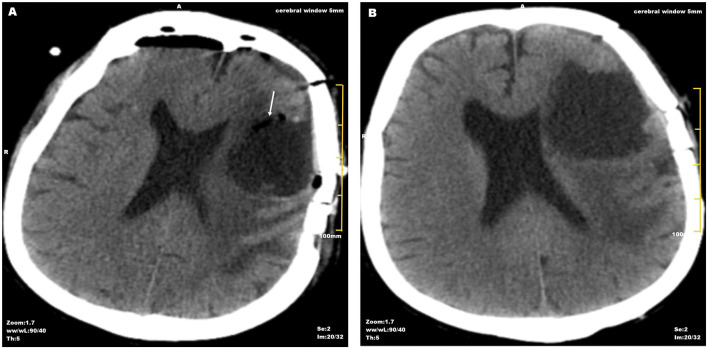
Postoperative computed tomography evolution following neurosurgical drainage. **(A)** Non-contrast CT on Day 15 showing surgical cavity with residual pneumocephalus (arrows) and mild effusion; midline structures centered. **(B)** CT on Day 25 demonstrating resolution of pneumocephalus and reduction in lesion size with decreased edema, indicating favorable response to therapy.

**FIGURE 4:**
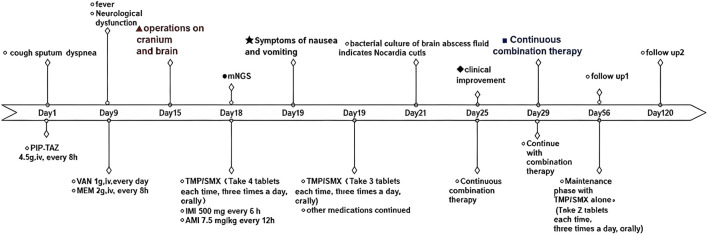
Timeline of antimicrobial therapy and clinical course. Chronological representation from Day 1 to Day 120. Abbreviations: PIP-TAZ, piperacillin-tazobactam; VAN, vancomycin; MEM, meropenem; TMP-SMX, trimethoprim-sulfamethoxazole (doses as TMP component); AMI, amikacin; IMI, imipenem. Key events: ▲ craniotomy and drainage; ● N. farcinica identification; ★ Grade 3 GI toxicity with TMP-SMX dose reduction; ◆ clinical improvement; ■ discharge; → transition to oral monotherapy.

By Day 25, fever resolved (36.4 °C) with partial neurological improvement, and imaging demonstrated significant reduction in abscess size and perilesional edema (Figure 3). The patient was discharged on Day 29 receiving TMP-SMX monotherapy (7.5 mg·kg^−1^·d^−1^); the detailed timeline of antimicrobial therapy is presented in Figure 4. At Day 120 follow-up, he remained clinically stable—neurologically intact, afebrile, and without cardiac symptoms—with no evidence of disease recurrence or progression. However, several limitations warrant acknowledgment: first, the patient did not undergo imaging follow-up after discharge due to local medical and economic constraints; second, this 120-day observation period remains shorter than the recommended 12-month surveillance for CNS nocardiosis, such that late recurrence cannot be definitively excluded. Long-term follow-up at 12 months is currently ongoing.

## 4 Discussion

This case illustrates the critical interplay between occupational lung disease and iatrogenic immunosuppression in predisposing to invasive nocardiosis. The superimposition of long-term corticosteroid therapy upon pneumoconiosis creates a synergistic immunodeficiency state that substantially amplifies infection risk (pooled OR 3.8, 95% CI 2.4–5.9) (Wang et al., 2023). Consistent with current IDSA/ESCMID guidelines recommending triple combination therapy for CNS nocardiosis in immunocompromised hosts (Bodilsen et al., 2024; Yetmar et al., 2025), we employed an initial regimen of imipenem, amikacin, and TMP-SMX.

However, the management of TMP-SMX dosing in this case highlights critical challenges when standard protocols prove intolerable. The observed clinical stabilization following dose de-escalation from 15 to 7.5 mg·kg^−1^·d^−1^ (TMP component) raises questions regarding linear dose-efficacy relationships. Nevertheless, we emphasize that this reduction was necessitated by life-threatening Grade 3 toxicity, representing a temporary risk-benefit adjustment rather than planned dose optimization.

Several confounding factors complicate interpretation of the therapeutic response. First, the lack of therapeutic drug monitoring (TDM) to confirm target steady-state concentrations (Css 100–150 μg/mL for TMP) (Trimethoprim-Sulfamethoxazole, 2024) represents a critical methodological limitation; we cannot exclude the possibility that higher tissue concentrations compensated for lower plasma doses, nor can we confirm adequate CNS penetration at the reduced dose. Second, phenotypic susceptibility testing confirmed trimethoprim-sulfamethoxazole sensitivity, but quantitative MIC data were not obtained, precluding MIC-guided dosing optimization. Third, concomitant corticosteroid tapering may have contributed to clinical improvement through immune reconstitution, independent of antimicrobial efficacy. Fourth, the possibility that clinical improvement resulted primarily from surgical drainage rather than antimicrobial therapy alone cannot be excluded. Finally, as an observational case study (n = 1), no inferential statistical analysis was possible, and the observed associations may not reflect causality.

Regarding antimicrobial selection, transient defervescence was achieved with meropenem monotherapy by Day 14; however, persistent neurological deficits and the severity of central nervous system infection necessitated transition to definitive combination therapy. While imipenem demonstrates superior *in vitro* activity against N. farcinica (MIC_90_ 2 vs. 8 mg/L) compared with meropenem (Hershko et al., 2023), the sustained clinical resolution observed in this case likely reflects synergistic effects of the triple regimen (imipenem, amikacin, and TMP-SMX) rather than single-agent superiority. This therapeutic shift was microbiologically justified by the presence of β-lactamase genes (*bla{FAR-1}* and *bla{FAR-1_1}*) in *N. farcinica* (Nathar et al., 2024), which confer intrinsic resistance to cephalosporins and certain carbapenems, thereby necessitating imipenem-based triple therapy for severe CNS infections.

This aligns with current consensus supporting combination therapy for severe CNS nocardiosis in immunocompromised hosts, though prospective validation remains lacking.

The integration of rapid diagnostics with targeted therapy likely contributed to the favorable outcome. mNGS enabled pathogen identification within 48 h, facilitating early targeted therapy and informed antimicrobial selection, contrasting sharply with conventional cultures requiring 7–14 days. This diagnostic-pharmacological synergy underscores the value of advanced diagnostics in rapidly progressive CNS infections.

Critical limitations preclude generalization of these findings. First, as noted, TDM was not performed, likely resulting in subtherapeutic plasma concentrations during maintenance therapy. Second, the reduced dose regimen (7.5 mg·kg^−1^·d^−1^) falls substantially below current guideline recommendations (10–15 mg·kg^−1^·d^−1^ for 6–12 months) and should not be interpreted as supporting routine low-dose maintenance therapy. Third, the 120-day follow-up remains insufficient to exclude late recurrence; 12-month radiological and clinical surveillance represents the standard of care. Fourth, no imaging follow-up was obtained due to patient constraints, precluding radiological confirmation of durable cure.

Furthermore, we acknowledge potential selection bias inherent in single-center, single-patient observations. The observed response might alternatively reflect: (i) delayed effects of initial high-dose therapy rather than efficacy of reduced maintenance dosing; (ii) lower virulence or higher intrinsic susceptibility of this specific N. farcinica isolate; or (iii) the favorable pharmacokinetic profile of imipenem and amikacin in CNS penetration, potentially compensating for suboptimal TMP-SMX exposure.

In conclusion, this case demonstrates the feasibility of temporary, toxicity-guided TMP-SMX dose reduction as a bridge strategy in exceptional circumstances characterized by life-threatening adverse events, provided intensive clinical monitoring is maintained. However, this approach represents a deviation from standard of care necessitated by clinical exigencies, not an endorsement of low-dose maintenance regimens. Optimal management remains guideline-concordant high-dose therapy with TDM-guided adjustments when available. Prospective studies with pharmacokinetic/pharmacodynamic profiling are urgently needed to define minimum effective exposures and validate TDM protocols for TMP-SMX in CNS nocardiosis.

## 5 Conclusion

Pneumoconiosis patients receiving corticosteroids are at elevated risk for disseminated N. farcinica CNS infection with high mortality. This case illustrates that in exceptional circumstances of severe, life-threatening drug intolerance, temporary TMP-SMX dose reduction with intensive clinical monitoring may serve as a bridge to complete therapy. However, we emphasize three critical caveats: (1) Guideline-concordant full-course therapy (TMP-SMX 10–15 mg·kg^−1^·d^−1^ of the TMP component for 6–12 months) remains the standard of care, and combination therapy was essential for initial disease control; (2) Therapeutic drug monitoring should be routinely implemented to ensure target concentrations (Css 100–150 μg/mL for the TMP component); (3) This case demonstrates feasibility of temporary reduction in exceptional toxicity, not efficacy of low-dose maintenance—durable cure remains unconfirmed at 120-day follow-up without imaging confirmation. Timely surgical drainage, early molecular diagnosis, and combination antimicrobial therapy facilitated initial clinical resolution in this high-risk host. Temporary dose modification should be reserved for exceptional cases with close longitudinal monitoring.

## Data Availability

The data supporting this study are available from the corresponding author upon reasonable request.

## References

[B1] Bodilsen J. D'Alessandris Q. G. Humphreys H. Iro M. A. Klein M. Last K. (2024). European society of clinical microbiology and infectious diseases guidelines on diagnosis and treatment of brain abscess in children and adults. Clin. Microbiology Infection The Official Publication Eur. Soc. Clin. Microbiol. Infect. Dis. 30 (1), 66–89. 10.1016/j.cmi.2023.08.016 37648062

[B2] Hershko Y. Levytskyi K. Rannon E. Assous M. V. Ken-Dror S. Amit S. (2023). Phenotypic and genotypic analysis of antimicrobial resistance in nocardia species. J. Antimicrobial Chemotherapy 78 (9), 2306–2314. 10.1093/jac/dkad236 37527397

[B3] Nathar S. Rajmichael R. Jeyaraj Pandian C. Nagarajan H. Mathimaran A. Kingsley J. D. (2024). Exploring Nocardia's ecological spectrum and novel therapeutic frontiers through whole-genome sequencing: unraveling drug resistance and virulence factors. Arch. Microbiol. 206 (2), 76. 10.1007/s00203-023-03799-z 38267747

[B4] Rafiei N. Peri A. M. Righi E. Harris P. Paterson D. L. (2016). Central nervous system nocardiosis in Queensland: a report of 20 cases and review of the literature. Med. Baltim. 95 (46), e5255. 10.1097/MD.0000000000005255 27861348 PMC5120905

[B5] Soueges S. Bouiller K. Botelho-Nevers E. Gagneux-Brunon A. Chirouze C. Rodriguez-Nava V. (2022). Prognosis and factors associated with disseminated nocardiosis: a ten-year multicenter study. J. Infection 85 (2), 130–136. 10.1016/j.jinf.2022.05.029 35654278

[B6] Trimethoprim-Sulfamethoxazole W. T. F. E. (2024). Expert consensus on the clinical application of therapeutic drug monitoring for trimethoprim-sulfamethoxazole. Chin. J. Infect. Chemother. 24 (05), 497–506. (in chinese). 10.16718/j.1009-7708.2024.05.001

[B7] Wang C. Sun Q. Yan J. Liao X. Long S. Zheng M. (2023). The species distribution and antimicrobial resistance profiles of Nocardia species in China: a systematic review and meta-analysis. Plos Negl. Trop. Dis. 17 (7), e0011432. 10.1371/journal.pntd.0011432 37428800 PMC10358964

[B8] Yetmar Z. A. Khodadadi R. B. Chesdachai S. Mchugh J. W. Clement J. Challener D. W. (2025). Trimethoprim-sulfamethoxazole dosing and outcomes of pulmonary nocardiosis. Infection 53 (1), 83–94. 10.1007/s15010-024-02323-9 38922564 PMC11825568

