# Supplementary material for: Toxicity-guided dose modification for disseminated Nocardia farcinica brain abscess in a patient with pneumoconiosis: a brief research report
Source: Front Pharmacol. 2026 Apr 9;17:1805920. doi: 10.3389/fphar.2026.1805920 (PMC13102607; doi:10.3389/fphar.2026.1805920)

Figure S1 Chest computed tomography findings at admission.

(A) Axial CT (lung window: width 1500 HU, level -600 HU) demonstrating diffuse nodular infiltrates, fibrotic foci, and cicatricial emphysema consistent with advanced pneumoconiosis.

(B) Same acquisition protocol, axial CT at lower lung level

Figure S1

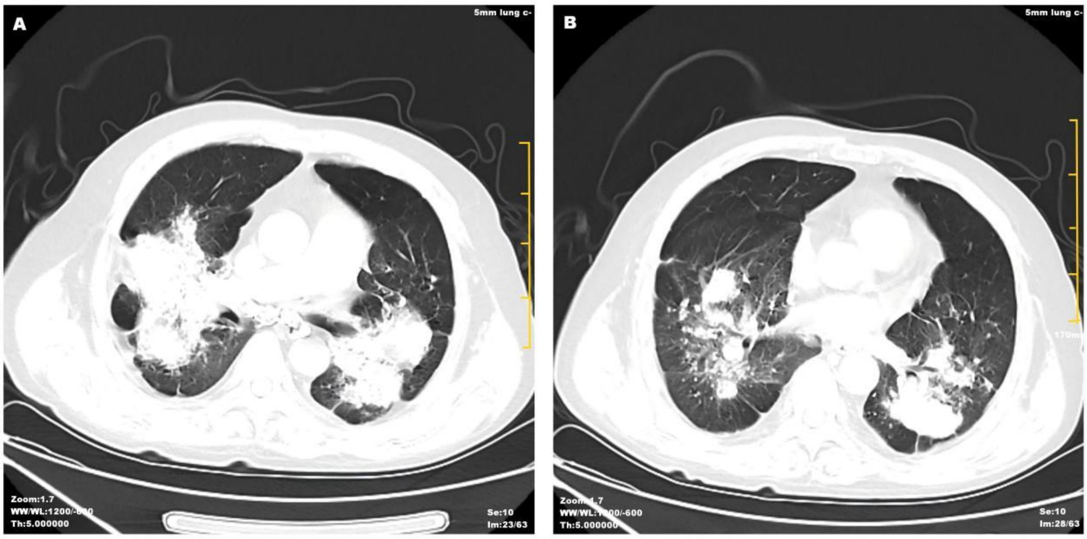

Supplement: Supplementary file 1 [file Supplementaryfile1.pdf]
